# Enlarged Prostate

**Published:** 1873-11

**Authors:** 


### ENLARGED PROSTATE.

An old man will come to you and say, "Doctor, I have trouble with my water; I void my urine very frequently; at night especially I am obliged to get up several times, and in the morning before breakfast I find that my bladder is irritable. Then, too, when I do try to pass my water only a little comes away, and that little comes in driblets, and seems not to be entirely under my control. The stream has no force; sometimes I have pain in my crotch, and I have often a most uncomfortable feeling just over the top of my bladder. Sometimes, too, it hurts me after I have ceased passing my water, and I make unavailing attempts to pass more." If you now inquire as to the nature of the urine passed he will often tell you that it is turbid, and contains a sticky, ropy mucus. Sometimes he will say that its smell is strong and offensive. Such is the history of one patient.

Another patient will come into your office, or, more likely you will be called to see him. As you approach him you detect an ammoniacal, urinous odor. If you look at his pantaloons you will often notice stains on the front, discolorations from the escape of urine. This patient is evidently very uncomfortable. You ask as to his symptoms, and he will mention some that I have already enumerated, but he will state in addition that although he has difficulty when he attempts to urinate, still there is a constant dripping of water from his penis.

Occasionally your patient may have attacks of partial or complete retention of urine. He can scarcely void any from his bladder. It often happens that such is his condition when you first make his acquaintance. His family doctor, perhaps, has suggested to him "paralysis of the bladder;" no such thing. Paralysis of the bladder, as has been well remarked by Sir Henry Thompson, is almost unknown, save as an accompaniment of cerebral or spinal lesion. Your patient is suffering from an accumulation of urine, and the dripping is due to over distension of the bladder, and to mechanical escape of some of its contents, and not to loss of the expulsive power of the organ. In the words of the high authority to whom I have already referred, "Involuntary micturition means retention, and not incontinence." And what has given rise to these symptoms I have endeavored to describe to you. What is the cause of this frequent micturition, or maybe of the fearful retention? In all probability, the exciting cause of these conditions will be found in just such a state of the parts as is before you in the dissection upon the table.

Gentlemen, I beg of you never to forget the appearances you now see. Imprint them upon your memory, and they will serve you in good stead hereafter. And how? In guiding you to the true treatment of these difficult cases of retention of urine, dependent upon prostatic enlargement, especially of its central portion.

This brings us to the *conduct* of your case. Your patient's story has suggested to you the idea of hypertrophy of the prostate. You make a digital examination by the rectum, and if the lateral lobes be increased in size, your diagnosis is confirmed. But in any event, you must relieve the over-distended bladder by the catheter. To do this, requires, ofttimes, the highest surgical skill; it is no work of chance. Hasty, rough manipulation will but add to your patient's misery; nay, more, it may ruin him, and may imperil your own fair fame.

Therefore you must act knowingly; and now look again at the dissection on the table; see, I will bring up the divided urethral walls, and as I do so, you will obtain a clear idea of the narrow tortuous channel through which your catheter must pass to gain entrance to the bladder. What sort of instrument will you choose? By some surgeons a silver catheter of large curve is recommended. I do not advise such an instrument; I do not mean to say that it may not answer in experienced and right skillful hands; but I am speaking to those who have, as yet, not acquired such skill. My present object is to suggest to you rather the management of your earlier cases. We will not, therefore worry too long with the metalic instrument; try it, if you like, but be careful in its use.

You have decided, on my advice, I think, to employ a flexible instrument. You may, if you please, make use of the ordinary English gum catheters, over-curved as suggested by Thompson; bent to its curve in warm water, and then thus fixed by being dipped in cold water. Here is such a catheter. But a better instrument even than this, is, I think, the one I show you now. Remember, once more, that sinuous canal of the urethra, running abruptly up and down at the will of the distorted prostate. The channel is almost as crooked as a swan's neck; and here gentlemen is an instrument, so yielding and flexible, and delicate, and soft, that it can be pushed forward almost with impunity, and almost, too, with a moral certainty of passing over the hillocks and through the valleys of the prostatic urethra. This catheter is the French, flexible probe-pointed catheter. Look at it, if you please, closely; you see that the beak is an inch and a half long, as flexible as a hair, and yet still retaining stiffness enough not to bend in the urethra. And here let me remind you that in the prostatic trouble we are speaking of the characteristic of the urethra is its tortuous course, rather than any great narrowing. Of course its diameter is not as large as in the normal state, but still the rigid contraction of a stricture is absent. The flexible probe-pointed catheter is not particularly suitable for the latter, whilst for the enlarged prostate it is, I think, exactly what you want.

How will you use this catheter? Suppose you are called in a hurry, in the middle of the night, to relieve an old gentlemen of retention. The whole household is in confusion; everybody, even the servants know what is the matter, and you are expected to succeed. It will not do to fail, nor will you if only you will have confidence in yourself. With such an instrument as I have shown you, you will in all probability succeed. You will usually find your patient in great distress, tossing on the bed and very clamorous. The first thing to be done is to put him in the proper position. Do not attempt to introduce your instrument from the side, but have your patient placed crossways in the bed with his buttocks on the edge, well supported by one or two pillows; the shoulders should be low. Let each of his legs be wrapped in a separate blanket, and be supported by a chair. Have his body covered so that he may not take cold.

Then stand between his legs, and with such a syringe as this throw two or three drachms of warm olive oil into the urethra, and by gentle rubbing on the urethra assist its passage downwards, or if you like you can use such a syringe as that in my hand, with a long catheter nozzle, which will carry the oil well downwards. Then elevate the penis so as to straighten the urethra; introduce a flexible probe-pointed catheter, of from number six to nine of the English scale, and carry it downwards. You will feel its point enter the tortuous channel of the prostatic urethra, and you will be sensible of the grasping resistance. Keep up a *gentle, continuous* pressure, and the chances are ten to one that the point of your instrument will glide into the bladder, and that a welcome flow of urine will result.

Sometimes the instrument will pass into the bladder, or reach its urethral orifice, and you may have no flow. This may depend upon the viscid character of the contents of the bladder. The urine may be charged with blood, or more frequently with thick ropy mucus, so glairy and tenacious, as to clog and block up the eyes of the catheter. I have often had this happen, and advise you to meet this difficulty by suction. Take your syringe, fit it to the projecting end of the catheter (here you see a conical pipe, which can be easily adjusted to catheters of all sizes), and then draw up the piston. If no urine follows, disconnect the syringe, refit it and repeat the manœuvre; usually, after one or two attempts, you will be gratified by a flow of urine. If the obstruction in the eyes of the catheter is very obstinate, I sometimes throw down a little warm water and suck it up. This will generally bring with it the occluding material. When the urine once begins to flow, there is not much after trouble.

This, then, is the method I advise you to adopt in treating prostatic retention; as you have seen, all that you will require will be a hard rubber penis syringe, and two or three French probe-pointed flexible catheters. Other methods there are of relieving this trouble. Many surgeons prefer the long silver instrument, while others employ with advantage the jointed silver catheter, devised by Dr. Squire, or this model, the vertebrated instrument of Dr. Sayre. All of these answer their purpose; I think, however, that greater skill is required in the use of the metallic instruments; and I am now speaking to you as beginners, who have yet to acquire your skill by experience. I am quite sure that you will find the course I have pointed out to you the easiest for yourself and the safest for your patients in your early professional life.

There is one matter yet to be considered. Let us suppose that you have drawn off your patient's water, and have relieved him for the time being. Shall you allow the instrument to remain *in situ,* or shall you take it away and trust to the chances of a re-introduction? The latter is usually my plan; in my experience the presence of a catheter in an already irritated urethra is hurtful; and I have seen it more than once give rise to great trouble and produce fresh and aggravated attacks of cystitis, which have led to most unfortunate results. I advise you, therefore, to withdraw the instrument and re-insert it as occasion demands. You may, if you like, on the principle of "letting well enough alone," make an exception in those cases where the passage is peculiarly difficult, and allow the catheter to rest in the urethra. I think, however, that you will find that with increasing skill will come a greater confidence and self-reliance on your part, and with them, I believe, better chances for your patients.

In the preceding remarks I have made no allusion to anæsthesia. I wish you, however, to understand that although I usually, in cases of retention, make the first attempt without the use of ether, I do not hesitate to resort to this agent if I experince much difficulty in introducing the catheter, or if the patient's urethra be very tender and irritable. Occasionally, too, the patient will be in a condition of demoralization, so much so as materially to interfere with your manipulations, until he is brought under the quieting influence of ether. You must bear in mind that catheterization for retention is always a delicate matter; and that in justice to yourself as well as to bring relief to your patient, you must give yourself the best chances in your operation; and these will, I think, be materially strengthened by the judicious use of the anæsthetic agent.

And now, gentlemen, I close my remarks as I began them, by begging you to photograph upon your minds the appearance of the three lobes of the prostate gland upon the table. That gland is typical of a great class of cases, and if you but remember its physiognomy in the future, it may serve to give dexterity to your hand, and comfort to many a patient in time to come.—*Philadelphia Hospital—Service of John M. Brenton, M. D.—Med. and Surg. Reporter.*

